# Protective Effects of Resveratrol against UVA-Induced Damage in ARPE19 Cells

**DOI:** 10.3390/ijms16035789

**Published:** 2015-03-12

**Authors:** Chi-Ming Chan, Cheng-Hua Huang, Hsin-Ju Li, Chien-Yu Hsiao, Ching-Chieh Su, Pei-Lan Lee, Chi-Feng Hung

**Affiliations:** 1 School of Medicine, Fu Jen Catholic University, New Taipei City 24205, Taiwan; E-Mails: m212092001@tmu.edu.tw (C.-M.C.); infection@cgh.org.tw (C.-H.H.); suk.ccsu@gmail.com (C.-C.S.); 2 Department of Ophthalmology, Cardinal Tien Hospital, Hsiendian, New Taipei City 23148, Taiwan; 3 Department of Internal Medicine, Cathay General Hospital, Taipei 10630, Taiwan; 4 Department of Chemstry, Fu Jen Catholic University, New Taipei City 24205, Taiwan; E-Mail: sakumanatsumi@gmail.com; 5 Department of Nutrition and Health Sciences, Chang Gung University of Science and Technology, Kweishan, Taoyuan 33303, Taiwan; E-Mail: mozart@gw.cgust.edu.tw; 6 Research center for Industry of Human Ecology, Chang Gung University of Science and Technology, Kweishan, Taoyuan 33303, Taiwan; 7 Graduate Institute of Applied Science and Engineering, Fu Jen Catholic University, New Taipei City 24205, Taiwan; 8 Department of Internal Medicine, Cardinal Tien Hospital, Hsiendian, New Taipei City 23148, Taiwan; 9 Slone Epidemiology Center, Boston University, Boston, MA 02215, USA; E-Mail: hlittlem@gmail.com

**Keywords:** resveratrol, UVA, retinal pigment epithelial (RPE) cells, age-related macular degeneration (AMD)

## Abstract

Ultraviolet radiation, especially UVA, can penetrate the lens, reach the retina, and induce oxidative stress to retinal pigment epithelial (RPE) cells. Even though it is weakly absorbed by protein and DNA, it may trigger the production of reactive oxygen species (ROS) and generate oxidative injury; oxidative injury to the retinal pigment epithelium has been implicated to play a contributory role in age-related macular degeneration (AMD). Studies showed that resveratrol, an abundant and active component of red grapes, can protect several cell types from oxidative stress. In this study, adult RPE cells being treated with different concentrations of resveratrol were used to evaluate the protective effect of resveratrol on RPE cells against UVA-induced damage. Cell viability assay showed that resveratrol reduced the UVA-induced decrease in RPE cell viability. Through flow cytometry analysis, we found that the generation of intracellular H_2_O_2_ induced by UVA irradiation in RPE cells could be suppressed by resveratrol in a concentration-dependent manner. Results of Western blot analysis demonstrated that resveratrol lowered the activation of UVA-induced extracellular signal-regulated kinase, c-jun-NH_2_ terminal kinase and p38 kinase in RPE cells. In addition, there was also a reduction in UVA-induced cyclooxygenase-2 (COX-2) expression in RPE cells pretreated with resveratrol. Our observations suggest that resveratrol is effective in preventing RPE cells from being damaged by UVA radiation, and is worth considering for further development as a chemoprotective agent for the prevention of early AMD.

## 1. Introduction

Oxidative injury and functional impairment of retinal pigment epithelial (RPE) cells may play an early and crucial role in the development of age-related macular degeneration (AMD) [1,2,3,4], one of the most common causes of severe visual loss in the elderly population in the developed world [5,6]. Exposure to ultraviolet (UV) A and short-wavelength visible radiation, even from natural environment, may induce the production of reactive oxygen species (ROS) and result in oxidative damage to RPE cells [3,7,8]. Previous studies have indicated that damage to the retina and RPE through photochemical mechanisms by free radical reactions is driven by photo-excited, endogenous chromophores through the cornea and lens [2]. The retina of a child is particularly susceptible to damage from UV exposure as the lens lacks the yellow pigment that prevents UV transmission [4]. As we age, the yellow pigment accumulates; however, oxidative damage increases, antioxidant capacity decreases and the efficiency of self-repairing systems deteriorate during the aging process. Loss of RPE cells, cataract formation, and retinal dysfunction may lead to visual impairment. In addition, cataract surgery may further worsen the situation by removing the natural lenticular UV and blue-light filter. Even though intraocular lenses (IOLs) usually have the UV and blue-light filters incorporated, the quality of the filters in different IOLs varies [9], and some UV and blue-light filtering IOLs still lack sufficient UVA protection [10].

Studies have shown that resveratrol (3,5,4'-trihydroxystilbene), a flavonoid found in red grapes and many fruits, exhibits antioxidant [11], anti-proliferative [12], anti-inflammatory [13] and chemopreventive [14] activities *in vitro.* Several potential health benefits, including reduced risk of cancer and heart disease, are also thought to be associated with the consumption of resveratrol [15,16]. Moreover, resveratrol has been reported to have antioxidant effects against hydrogen peroxide-induced oxidative stress [17] and acrolein-induced cytotoxicity in human RPE cells [18]. However, there have been few studies on the protective effects of resveratrol against UVA-induced damage, and the underlying mechanism of its effects is still unknown.

In this study, we investigated the protective effects of resveratrol against UVA-induced decrease in RPE cell viability and the possible mechanisms involved, including the inhibition of UVA-induced intracellular hydrogen peroxide (H_2_O_2_) production, mitogen-activated protein kinase (MAPK) activation, and cyclooxygenase-2 (COX-2) expression.

## 2. Results

### 2.1. Resveratrol Has no Cytotoxicity on ARPE19 Cells

**Figure 1:**
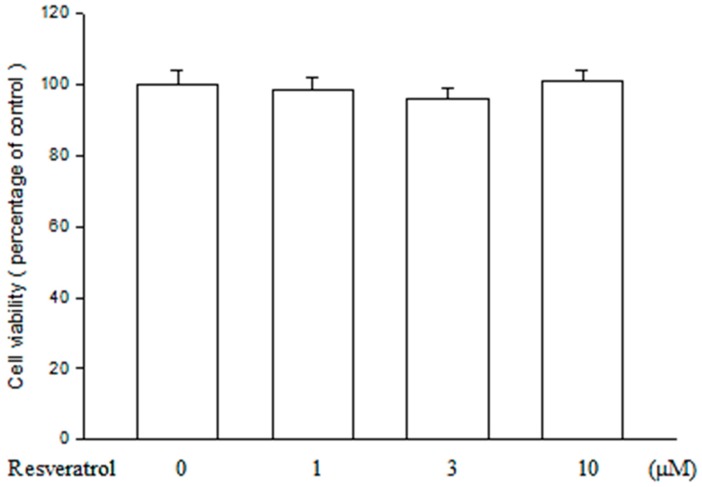
Resveratrol is not cytotoxic to ARPE19 cells. After ARPE19 cells were treated with different concentrations of resveratrol for 24 h, cell viability was assessed using 3-(4,5-Dimethylthiazol-2-yl)-2,5-Diphenyltetrazolium Bromide (MTT) assay. No significant cytotoxicity of resveratrol to the cells was found when comparing to those not being treated. The results are expressed as percentage of control and represented by mean ± standard error (SE) (*n* = 3).

Before the experiment, cell viability assay was used to evaluate the toxic effect of resveratrol on ARPE19 cells. As shown in Figure 1, no significant change in cell viability was found after ARPE19 cells being treated with resveratrol in various concentrations between 1 and 10 μM. The data indicate that resveratrol is safe for ARPE19 cells at the concentrations used in this study.

### 2.2. Resveratrol Reduced UVA-Induced Decrease in Cell Viability

**Figure 2:**
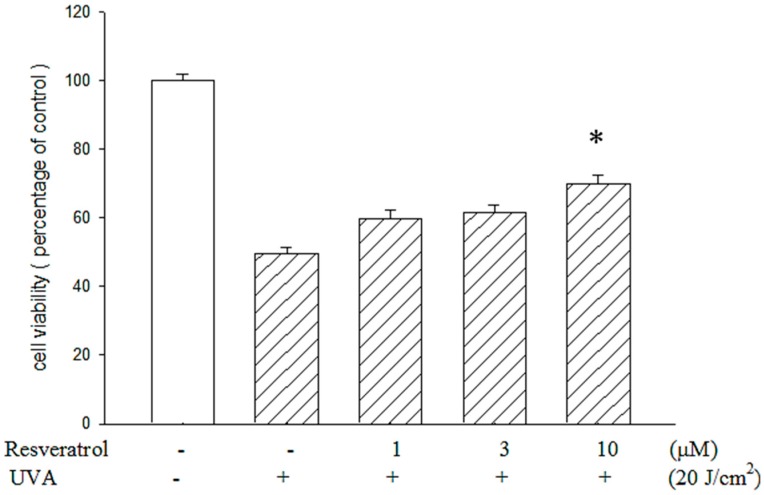
Protective effect of resveratrol on ARPE19 cells against UVA radiation exposure. MTT assay showed that the cell viability of ARPE19 cells against UVA radiation (20 J/cm^2^) was protected by resveratrol in a dose-related manner. The results are expressed as a percentage of the control group to which neither resveratrol nor UVA was given. The results are represented by mean ± SE (*n* = 3). * indicates that it is significantly different from UVA-exposed cells without resveratrol pretreatment (*p* < 0.05).

Cell viability assay showed that the viability of ARPE19 cells dropped after UVA exposure; the decrease was reduced by pretreating the cells with resveratrol at the concentrations of 1, 3 and 10 μM (Figure 2). Particularly at the concentration of 10 μM, the survival rate of RPE cells pretreated with resveratrol was significantly higher (*p* < 0.05) than those without treatment; approximately 75% of pretreated cells remained viable upon UVA exposure. These observations indicate that resveratrol is effective in the prevention of UVA-induced ARPE19 cell damage.

### 2.3. Resveratrol Lessened UVA-Induced H_2_O_2_ Production

**Figure 3:**
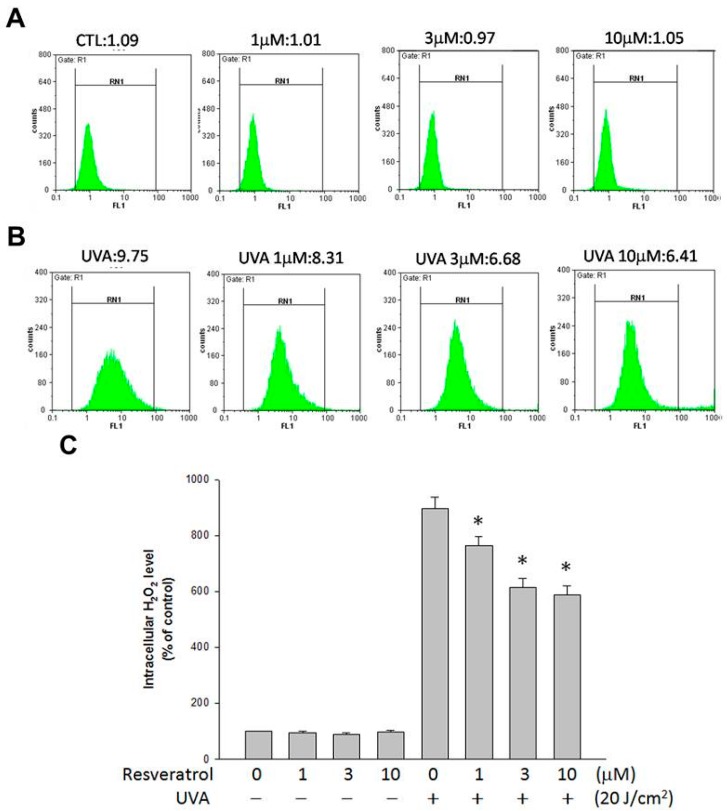
The amount of H_2_O_2_ production in ARPE19 cells after UVA radiation was suppressed by resveratrol. Representative histograms of cell counts *versus* fluorescence intensity indicate the amount of intracellular H_2_O_2_ in ARPE19 cells pretreated with PBS and different amounts of resveratrol for 24 h: (**A**) before; and (**B**) after being exposed to 20 J/cm^2^ of UVA radiation; (**C**) Quantitative analyses of intracellular H_2_O_2_ in ARPE19 cells presented as percentage of control and represented by mean ± SE of three independent experiments. * indicates that the different between UVA-exposed cells with and without resveratrol pretreatment is significant (*p* < 0.05).

Flow cytometric analysis was used to determine whether resveratrol could inhibit UVA-induced intracellular H_2_O_2_ production. The amount of intracellular H_2_O_2_ in ARPE19 cells was measured using DHR 123, a dye that has been shown to react with H_2_O_2_ in the presence of peroxidase and is used for the detection of intracellular H_2_O_2_. Without exposing to UVA, the amount of intracellular H_2_O_2_ was not affected by the treatment of resveratrol (Figure 3A). However, intracellular H_2_O_2_ production increased about nine fold in UVA-exposed cells over unexposed control cells (Figure 3A,B); the increase was lessened when the cells were pretreated with resveratrol in a concentration-dependent manner (Figure 3B). Treatment with 1, 3 and 10 μM of resveratrol significantly inhibited intracellular H_2_O_2_ production when compared with the UVA-irradiated culture without resveratrol treatment (Figure 3C; *p* < 0.05), which indicates that resveratrol can prevent intracellular H_2_O_2_ production when ARPE19 cells are challenged with UVA irradiation.

### 2.4. Resveratrol Suppressed UVA-Induced MAPK Activation

**Figure 4:**
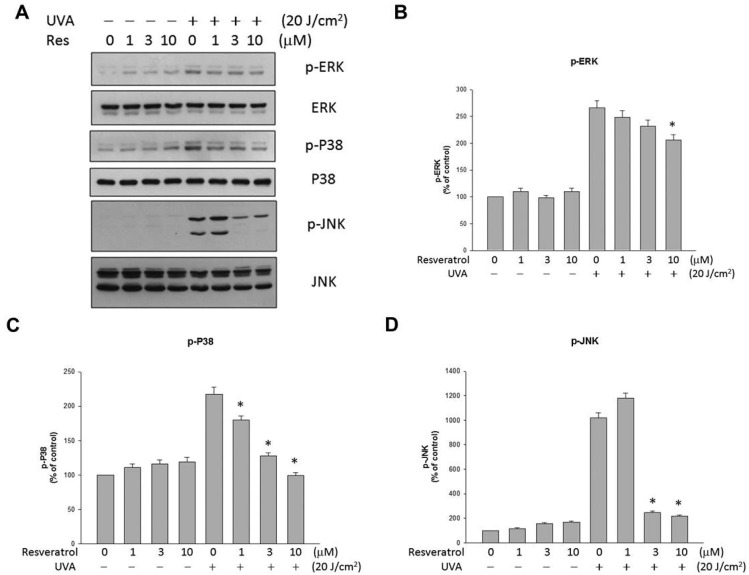
Resveratrol suppressed the production of UVA-induced ERK, p38 and JNK phosphorylation. (**A**) Western blot analysis of ARPE19 cells after they were preincubated with resveratrol for 2 h and exposed to UVA irradiation (20 J/cm^2^). The changes in phosphorylated ERK, p38 and JNK expression were evaluated; (**B**–**D**) Quantitative results of the western blot analyses expressed as percentage of control and represented by mean ± SE of three independent experiments to quantify and average the results. * indicates that the result is significantly different (*p* < 0.05) from UVA-stimulated cells without resveratrol pretreatment (the fifth bar).

Since UVA irradiation activates MAPK phosphorylation [7,19], we evaluated the effect of resveratrol on the levels of ERK1/2, p38 and JNK phosphorylation in ARPE19 cells. Figure 4 shows that the levels of ERK1/2, p38 and JNK phosphorylation were elevated in UVA-irradiated ARPE19 cells, and the increases could be significantly lowered with the treatment of resveratrol. Reprobing of the immunoblots with antibodies raised against total ERK1/2, JNK and p38 demonstrated the even loading of each sample (Figure 4B–D, lower panels). Our results demonstrate that resveratrol affects MAPK activation.

### 2.5. Resveratrol Lowered UVA-Induced COX-2 Expression

**Figure 5:**
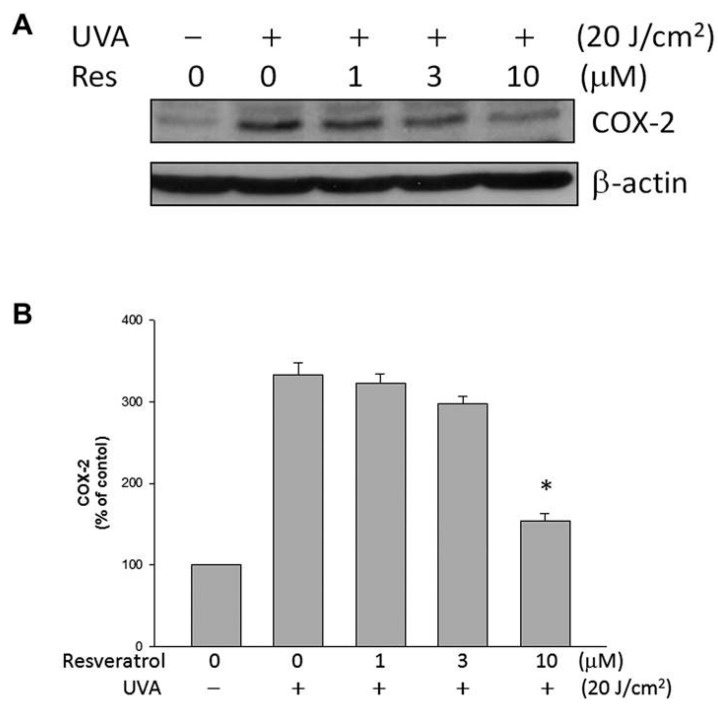
Resveratrol lowered UVA-induced COX-2 expression. (**A**) Levels of COX-2 expression in ARPE19 cells evaluated by western blot analysis; (**B**) Quantitative results of western blot analyses expressed as percentage of control and represented by mean ± SE of three independent experiments to quantify and average the results. * indicates that the different is significant (*p* < 0.05) when compared to UVA-irradiated cells without resveratrol pretreatment.

COX-2 expression in ARPE19 cells was studied as a possible protective mechanism of resveratrol on ARPE19 cells against the damaging effects of UVA radiation. UVA radiation triggered the increase of COX-2 expression in ARPE19 cells; treatment with resveratrol reduced the increase (Figure 5). The result suggests that inflammatory process, generally associated with an elevated level of COX-2 expression, may be correlated with the UVA-induced decrease in ARPE19 cell viability, and the reduction in COX-2 expression suggests that resveratrol may be able to suppress the inflammatory process after UVA exposure.

## 3. Discussion

Resveratrol belongs to the stilbene family of compounds, which are characterized by two aromatic rings joined by a methylene bridge. It exists in two isoforms, the *trans* and the *cis* isomers. *Trans*-resveratrol, the primary form in natural plants, has a greater biological activity, and has been extensively investigated [20]. Resveratrol was reported to reduce oxidation and cell proliferation by inhibiting ERK in retinal pigment epithelial cells [21]. Resveratrol has also been found to significantly reduce oxidative damage of the phagocytic function of human RPE R-50 cells with the underlying mechanisms linked to the activity of large-conductance calcium-activated potassium channels (BK(Ca) channels) in RPE cells [22]. In addition, resveratrol suppresses platelet-derived growth factor-BB-induced retinal pigment epithelial cell migration [23], and protects against UVA-mediated inhibition of the phagocytic function of human retinal pigment epithelial cells via BK(Ca) channels [24]. However, the mechanism behind the protective effects of resveratrol on RPE cells from being damaged by UVA radiation has remained unclear. Our study evaluated the ability and the possible mechanism of resveratrol in protecting against UVA-induced injury in RPE cells. We observed that resveratrol reduced the UVA-induced decrease in cell viability; lowered H_2_O_2_ production, MAPK activation and the expression of COX-2 in RPE cells (Figure 3 and Figure 5). These observations suggest that resveratrol may prevent UVA-induced RPE cells death through anti-oxidative and anti-inflammatory activities, and may be helpful in the prevention of early AMD.

UVA is by far the most abundant solar UV radiation that reaches the surface of earth. Even though UVA is weakly absorbed by DNA, and it is not acting like UVB that can be absorbed by nucleic acids and induces genotoxic damage to DNA directly, UVA excites other endogenous chromophores, and generate various reactive oxygen species (ROS) in cells. UVA radiation is known to be able to generate oxidative damage, single- and double-strand breaks, produce secondary photoreactions, damage DNA by indirect photosensitizing reactions, and induce photoproducts such as 8-oxo-7 and 8-dihydro-2'-deoxyguanosine (8oxoG) [25,26]. As UVA is a strong generator of oxidative stress and hydrogen peroxide can increase the apoptosis of RPE cells, we studied the UVA-induced H_2_O_2_ production in RPE cells. We found that UVA induced H_2_O_2_ production in RPE cells, and it could be reduced by incubating RPE cells with resveratrol. The decrease of H_2_O_2_ production may have contributed to the protective effects of resveratrol against the UVA-induced decrease in RPE cell viability; however, we also found that UVA-induced H_2_O_2_ production was not totally abolished by resveratrol treatment, which may have prevented the complete elimination of the decrease in cell viability induced by UVA (Figure 2 and Figure 3).

Activation of MAPK is essential for ROS-induced apoptosis in RPE cells [27]. UV irradiation has been reported to upregulate MAPK signaling pathways [28] and MAPK phosphorylation, including those of ERKs, JNKs and p38 kinase [7,28,29]. JNK and p38 activation are essential for UV-induced apoptosis, but activation of ERK, NF-κB, PI3K/Akt, and the mammalian target of rapamycin (mTOR) complex can be the surviving pathways against UV-induced death signaling [30,31]. However, ERK1/2 is also involved in oxidative-stress-induced VEGF upregulation [32], which may be related to choroidal neovascularization. Our study found that resveratrol decreased UVA-induced ERK1/2, JNK and p38 activation and reduced oxidative stress in the RPE cells. At the same time, the inhibition of JNK and p38 may have contributed to the protective effect of resveratrol against the UVA-induced decrease in cell viability.

Several studies have revealed that COX-2 expression can be induced in some types of skin cells by the irradiation of UVA [29,33,34]. Our previous study showed an increase in the expression of COX-2 in UVA-irradiated RPE cells [7]. COX-2 is thought to play a major role in angiogenesis by inducing the synthesis of prostaglandins, which may invoke signaling cascades to perform crosstalk and synergistic effect with diverse signaling pathways including vascular endothelial growth factor (VEGF)-signaling that stimulate the expression of proangiogenic factors [35,36]. It has been shown that selective COX-2 inhibitors attenuate choroidal neovascularization formation in an animal model of angiogenesis [37,38,39], and reduce choroidal neovascular membrane formation in cyclooxygenase-2 null mice [40]. The expression of COX-2 in human choroidal neovascular membranes implicates COX-2 in AMD pathogenesis [41]. In this study, we found that resveratrol can decrease COX-2 expression, which suggests that resveratrol has protective effect against the formation of choroidal neovascularization. Furthermore, MAPK activation is associated with COX-2 expression in several cell types [42]. As a result, the suppression of MAPK phosphorylation by resveratrol may contribute to the reduction of COX-2 expression.

In summary, our study demonstrated the protective effects of resveratrol on RPE cells against UVA-induced damages through suppressing UVA-induced H_2_O_2_ production, MAPK activation, and COX-2 expression; these inhibitory activities may have contributed to the increase in cell viabilities after UVA exposure. Our observations suggest that resveratrol may act as a suppressing agent for the prevention of ROS or UVA-induced ocular disorders. Lastly, this study may also provide a foundation for future studies in relevant animal models or other systems to evaluate the possible protective effect of resveratrol against AMD, and it is worth to repeat this model with different RPE cell line.

## 4. Experimental Section

### 4.1. Materials

Adult human retinal pigment epithelial cells (ARPE19), purchased from Food Industry Research and Development Institute (Hsinchu, Taiwan), were used to evaluate the protective effect of Resveratrol against the damage of UVA irradiation. Resveratrol was purchased from Sigma Chemical Co., (St. Louis, MO, USA). Other biological and chemical materials used were purchased from the following companies: 3-(4,5-dimethylthiazol-2-yl)-2,5-diphenyltetrazolium bromide (MTT), aprotinin, leupeptin, phenylmethylsulfonyl fluoride (PMSF), sodium fluoride (NaF), and sodium orthovanadate were also purchased from Sigma Chemical Co. (St. Louis, MO, USA); anti-p38 and anti-phospho-c-jun-NH_2_ terminal kinase (JNK) were purchased from Cell Signaling Technology (Beverly, MA, USA); anti-JNK, anti-extracullar signal-regulated kinase (ERK)1/2, and anti-phospho-p38 were purchased from R&D System, Inc., (Minneapolis, MN, USA); antirabbit-HRP, antigoat-HRP, anti-phospho-ERK1/2, and anti-COX-2 were purchased from Santa Cruz Biotechnology (Santa Cruz, CA, USA); and anti-dihydrorhodamine 123 (DHR 123) was purchased from Molecular Probes (Eugene, OR, USA).

### 4.2. Cell Preparation and UV Radiation

Adult human retinal pigment epithelial cells (ARPE19) were incubated in Dulbecco’s Modified Eagle’s Medium/Nutrient Mixture F-12 (DMEM/F12) supplemented with 10% fetal calf serum (GibcoBRL, Invitrogen Life Technologies, Carlsbad, CA, USA), 100 units/mL penicillin, and 100 μg/mL streptomycin (Sigma Chemical Co.) in a humidified incubator at 37 °C with 5% CO_2_; 24-well plates, 6-well plates (Costar, Cambridge, MA, USA), and 6 cm culture dishes (Costar) were used for the culture for cell viability assays, flow cytometric analysis, and western blot analysis respectively. Cells reaching a 90%–95% of confluence were starved and synchronized in serum-free DMEM for 24 h before further analysis. Before UV radiation, the cells were divided into control groups and experimental groups that were either not being treated or being treated with various concentrations of resveratrol for 2 h. Afterward, the cells prepared for flow cytometric analysis were further incubated in DMEM/F12 solution with 10 μg/mL of DHR 123 added for another 30 min after being washed with PBS and DMEM/F12. UVA irradiation using a Bio-Sun system illuminator from VilberLourmat (Marne-la-ValléeCedex 1, France) was then applied with the culture mediums being replaced by 300 μL/well, 500 μL/well and 1000 μL/dish phosphate-buffered saline (PBS; 137 mM NaCl, 10 mM Phosphate, 2.7 mM KCl, and pH of 7.4) respectively. UVA radiation was supplied by a closely spaced array of four UVA lamps that delivered uniform irradiation at a distance of 10 cm. The UVA lamps emit ultraviolet rays between 355 and 375 nm, with peak luminosity at 365 nm. It took approximately 74–80 min to attain (irradiance: 4.2–4.5 mW/cm^2^) the target UVA irradiation dose, 20 J/cm^2^. With a programmable microprocessor, the Bio-Sun system stopped the UV light emission automatically when the energy reached the programmed energy (range of measure: 0–99,999 J/cm^2^). Thereafter, the cells were treated differently according to different analytic groups for further analysis.

### 4.3. Cell Viability Assays

After UVA exposure, PBS- or resveratrol-pretreated cells were incubated for an additional 24 h with fresh DMEM/F12 containing resveratrol. Viability of the cells was then measured using 0.5 mg/mL MTT in DMEM/F12 after a brief wash with the medium. While mitochondrial dehydrogenases metabolized MTT to a purple formazan dye that can be analyzed photometrically at 550 nm, the absorbance, which is proportion to the quantity of living metabolically active cells, was measured to indicate cell viability.

### 4.4. Flow Cytometric Analysis

Through trypsinization and centrifugation, cell pellets were collected after UVA exposure; they were then resuspended in 1 mL of PBS. Intracellular H_2_O_2_ was being analyzed immediately by the PartecCyFlow ML flow cytometer (Partech GmBH, Munster, Germany) at excitation wavelengths of 488 nm and emission wavelengths of 525 nm. Fluorescence signals of 10,000 cells were collected to calculate the mean fluorescence intensity of a single cell.

### 4.5. Western Blot Analysis

After being washed with PBS twice, RPE cells, either exposed to UVA irradiation or not, were lysed in freshly prepared radioimmunoprecipitation assay buffer containing 17 mM Tris-HCl, pH 7.4, 50 mM NaCl, 5 mM EDTA, 1 mM NaF, 1% Triton X-100, 1% sodium deoxycholate, 0.1% SDS, 1 mM sodium orthovanadate, 1 mM PMSF, and 1 μg/mL aprotinin and leupeptin. After sonication, the lysate was centrifuged (14,000× *g* for 10 min at 4 °C), and the supernatant was removed. The protein content was then quantified by a Pierce protein assay kit (Pierce, Rockford, IL, USA). After the total protein was separated by electrophoresis on 8% SDS-polyacrylamide gels, the proteins were electroblotted onto polyvinylidene fluoride (PVDF) membranes and probed using specific antibodies. Immunoblots were then detected by enhanced chemiluminescence (Chemiluminescence Reagent Plus, NEN, Boston, MA, USA) to determine the level of ERK, JNK, p38 and COX-2 expression.The PVDF membrane was stripped at 60 °C for 30 min with a stripping buffer that contained 62.5 mM Tris-HCl, pH 6.7, 2% SDS, and 100 mM β-mercaptoethanol when necessary.

### 4.6. Data Analysis

All data were analyzed with SigmaPlot for Windows (Version 10.00). Unless otherwise indicated, data are expressed as mean ± standard error (SE). Comparison of the mean survival rates of cells exposed to UVA radiation with and without 10 µM resveratrol was made by using the unpaired, two-tailed Student *t*-test. We consider *p*-values <0.05 to be statistically significant.
